# A Serious Game (“Fight With Virus”) for Preventing COVID-19 Health Rumors: Development and Experimental Study

**DOI:** 10.2196/45546

**Published:** 2024-02-26

**Authors:** Shuo Xiong, Long Zuo, Qiwei Chen, Zhang Zeliang, Mohd Nor Akmal Khalid

**Affiliations:** 1 Philosophy and Social Sciences Laboratory of Big Data and National Communication Strategy Huazhong University of Science and Technology Wuhan China; 2 School of Information Engineering Chang’an University Xi'an China; 3 School of Journalism and Information Communication Huazhong University of Science and Technology Wuhan China; 4 School of Information Science Japan Advanced Institute of Science and Technology Ishikawa Japan; 5 School of Computer Science Universiti Sains Malaysia Georgetown Malaysia

**Keywords:** serious game, COVID-19, health rumor, game communication, game TCP model, Transmission Control Protocol, gaming, misinformation, disinformation, rumor, health communication, false information, elder, older adult

## Abstract

**Background:**

Health rumors arbitrarily spread in mainstream social media on the internet. Health rumors emerged in China during the outbreak of COVID-19 in early 2020. Many midelders/elders (age over 40 years) who lived in Wuhan believed these rumors.

**Objective:**

This study focused on designing a serious game as an experimental program to prevent and control health rumors. The focus of the study was explicitly on the context of the social networking service for midelders/elders.

**Methods:**

This research involved 2 major parts: adopting the Transmission Control Protocol model for games and then, based on the model, designing a game named “Fight With Virus” as an experimental platform and developing a cognitive questionnaire with a 5-point Likert scale. The relevant variables for this experimental study were defined, and 10 hypotheses were proposed and tested with an empirical study. In total, 200 participants were selected for the experiments. By collecting relevant data in the experiments, we conducted statistical observations and comparative analysis to test whether the experimental hypotheses could be proved.

**Results:**

We noted that compared to traditional media, serious games are more capable of inspiring interest in research participants toward their understanding of the knowledge and learning of health commonsense. In judging and recognizing the COVID-19 health rumor, the test group that used game education had a stronger ability regarding identification of the rumor and a higher accuracy rate of identification. Results showed that the more educated midelders/elders are, the more effective they are at using serious games.

**Conclusions:**

Compared to traditional media, serious games can effectively improve midelders’/elders’ cognitive abilities while they face a health rumor. The gameplay effect is related to the individual’s age and educational background, while income and gender have no impact.

## Introduction

### Background

In recent years, the arbitrary spreading of health rumors in mainstream social media on the internet has increasingly gained the attention of the public and raised concerns [1]. For health rumor researchers, a common concern is to propose a feasible and effective prevention and control program for current rampant rumors [2,3]. Furthermore, to prevent and control the spread of such rumors, it is necessary to strengthen the public’s health knowledge to judge and identify the rumors [4]. The concept of a rumor involves a form of statement whose veracity cannot be quickly or ever confirmed. Generally, we have a “dream rumor” and a “bogie rumor,” the former reflecting public desires and wished-for outcomes and the latter hiding some special purpose by somebody, both of them largely occurring during the early COVID-19 epidemic in Wuhan, China [4]. The traditional way of countering rumors often relies on media refutation, which can only be described as a “Band-Aid” solution. To fundamentally prevent and control COVID-19–related rumors and enhance the public's ability to resist them, we need to find a form of “information vaccine.” Therefore, we chose serious games as a “vaccine” in this context.

The purpose of a serious game is to help people acquire knowledge by playing games. Serious games involve solving problems and studying via careful and thoughtful game ideas [5], while considering characteristics beyond gameplay (eg, purpose and scope [6]). In addition, game elements are used to improve information processing and identify relevant information, which is consistent with the purpose of health rumor prevention research [7]. COVID-19–related rumors are based on the content of serious games to experiment with health rumor prevention, mainly using the control variable method and the analysis-contrast method to apply serious game learning to health rumor prevention research. This paper explores how to help people acquire knowledge of health rumors and health commonsense from the prevention experiment using the relaxed approach of serious games [8].

Previously, serious games have provided a platform for education and business use. For instance, behavioral interventions can be carefully tested and designed to reduce risk-taking behaviors [9], where transmission risks and the usefulness of pandemic-like simulations were demonstrated in the laboratory to be safely and ethically comprehended at the initial state of a health crisis. In addition, other studies prove that serious games are used to accommodate informational and communication complexities in early warning disaster management to simulate and test how public information from social media is used in emergency operation centers to make (protective and communicative) decisions based on levels of trust, usefulness, and completeness [10,11]. Therefore, serious games as an “information vaccine” have certain feasibility, and this paper also explored this issue. Nevertheless, the prevention and control of health rumors have rarely been considered in the context of the social networking service (SNS) for elderly users.

### Serious Games

Why are serious games chosen as a solution? Serious games refer to those electronic games whose main content is used for knowledge and skill development, professional training, and spreading culture. They are widely used in many fields. Compared to the limitations and congenital deficiencies of some communication models of traditional media, serious games have become an effective tool to address many social problems, because of their fast speed, wide range, and interactivity [12].

Abt [5] first defined the concept of serious games as follows: “These games have an explicit and carefully thought-out educational purpose and are not intended to be played primarily for amusement.” Later, Sawyer [8], in his white paper titled “Serious Game 2 Initiative,” redefined the concept of a serious game as being an entertainment game with nonentertainment goals. Several variants of the concept have also been proposed. Michael and Chen [13] defined serious games as games that educate, train, and inform. Meanwhile, Zyda [14] defined serious games as a mental contest played with a computer following specific rules. This situation led some analysts to describe serious games as the next wave of technology-mediated learning [15]. Although there is no single definition of the serious game concept, all the proposed definitions convey the same idea: using games to teach or transmit something [16].

Serious games are present in many areas. Westera et al [17] argued that serious games open up many new opportunities for learning complex skills, especially in the education and training domains [12,18-20]. Moreover, Yusoff et al [21] and Crookall [22] argued that good computer games are an excellent example of modern educational theory and that establishing simulation-based serious games as a discipline is a crucial endeavor that could benefit many other related disciplines.

Some early studies were systematically outlined by Connolly et al [23]. For instance, Ziebarth et al [18] and Diehl et al [19] adopted serious games to develop a prototype for the training and education of health students. Some scholars have emphasized the role of serious games in highly specialized skill acquisition (ie, drilling operation [24], mitigation of student dropout [25], improving the command performance of pilots [26]) and education (ie, medical surgery) [27,28], while providing the means to influence cognition and motivational driver [29].

Serious games are also being applied to pass on knowledge or expertise, which can be adopted for various purposes (ie, rehabilitation, psychotherapy, and brain disorders [30-32]). Sometimes, a serious game can also be used to increase risk awareness in the working area of the manufacturing floor [33]. The review by Abd-Alrazaq et al [34] showed that tools such as serious games are usable but are not replaceable options for rehabilitation and clinical intervention where long-term effects are required. Another review by Krath et al [33] revealed that serious games have also incorporated many theoretical foundations relevant to 3 significant landscapes: behavior, learning, and affect-motivation.

In research related to midelders/elders, several studies have demonstrated the potential of serious games to promote physical activity among older adults. For example, a randomized controlled trial conducted by Fu et al [35] found that a 6-month program of exergaming (exercise using video games) significantly improves the physical function of older adults. Similarly, a study by Jiménez-Pavón et al [36] showed that exergaming increases physical activity and cognitive performance in older adults with mild cognitive impairment.

Serious games have also been used to enhance cognitive training and disease management among older adults. For instance, a study by Anguera et al [37] found that cognitive training through a video game improves cognitive control in older adults. Additionally, a systematic review by Loerzel et al [38] indicated that serious games have the potential to improve self-management and quality of life among older adults with chronic diseases.

In COVID-19–related research, several studies have investigated the potential of gamification and serious games in promoting physical activity during the COVID-19 pandemic. For example, a study by Hall et al [39] proposed a project at a hospital’s senior health center in Canada to discuss how health care can be addressed using serious games among middle-aged and older adults during the pandemic [39]. The study found that the game was effective in increasing physical activity levels and improving self-efficacy. Lau et al [40] demonstrated the potential use of serious game to improve physical activity, cognitive training, and mental health among the aging population during COVID-19 in Hongkong.

Similarly, a study by Suppan et al [41] developed a serious game designed to promote safe behaviors for infection prevention and control (IPC), with a specific focus on COVID-19 among health care workers (HCWs) and other hospital employees. Another study by Ferreira et al [42] explored the potential of gamification in promoting hand hygiene among HCWs during the pandemic. The study found that the game was effective in increasing hand hygiene compliance among the participants [42].

Overall, gamification and serious games have emerged as a promising tool to promote physical activity and health and well-being during the COVID-19 pandemic. These technologies have the potential to support health promotion initiatives and encourage people to adopt healthy behaviors in a fun and engaging way. Therefore, we believe serious games can also solve the issue of COVID-19–related rumors that existed among Chinese midelders/elders.

### Health Rumor Analysis

Zhang et al [1] investigated all 453 features of health rumor data collected from a definitive online reference in China. A logistic regression model was adopted to determine the contribution of such features to true and false health rumors. There were measurable differences between true and false health rumors, where the length of a headline or statement and the presence of pictures were negatively correlated with the probability that a rumor was true. Meanwhile, a rumor was more likely to be true if it contained elements such as numbers, source cues, and hyperlinks. They also found that the dread health rumor is more likely to be true than a wishful one. Meanwhile, Chua and Banerjee [4] conducted a study on health rumors from 2015 to 2018. Users’ trust in online health rumors was investigated using 2 factors: length and presence of an image. Additionally, 2 types of rumors were studied: pipe-dream rumor, which offers hope, and bogie rumors, which instill fear. A total of 102 people participated in the experiment, where the finding suggested that pipe-dream rumors are trusted when they are short and do not contain images, while bogie rumors are trusted when they are long and contain images.

Subsequently, Chua and Banerjee [3] investigated the role of epistemic belief in affecting internet users’ decision to share online health rumors. The study focused on the characteristics of rumors—true or false, textual or pictorial, dread or wishful—shaping the decision-making among epistemologically naive and robust users separately. The study showed that epistemologically naive individuals are likelier to share online health rumors than epistemologically robust individuals. In addition, epistemologically robust participants were more likely to share textual rumors than pictorial ones. However, there were no differences between true and false rumors (or between dread and wishful rumors) among either epistemologically naive or robust participants. Meanwhile, Wu [43] modeled factors that predicted fake news sharing during the COVID-19 health crisis. Results showed that informational dependency and social dependency engender both positive and negative cognitive states, namely perceived information timeliness, perceived socialization, and social overload, which then invoke positive and negative affects. Considering that SNS dependency affects information-seeking behavior, it is important for individuals to be exposed to as much accurate information as possible and to build up rational communication against the spread of false rumors.

Ji et al [44] explored factors that influence people’s engagement in scientific rumormongering of genetically modified (GM) food on the Chinese social media platform Sina Weibo at both the group and the individual level. In total, 9070 posts about GM food were obtained from 1 million users. Analysis using logistic regression of the effect of peer influence did not find that users would depend on their friendship network to spread rumors. Instead, results revealed that people with negative attitudes toward GM food and who are social media extroverts (ie, celebrities) are more likely to spread rumors. In contrast, social reputation did not influence the spread of rumors, overwhelming the voices of the scientific community and negatively influencing public attitudes and behaviors.

Meanwhile, Hui et al [45] conducted a study on the spread mechanism of rumors on social network platforms during COVID-19 and considered education as a control measure against the spread of rumors. A novel epidemic-like model was established to characterize the spread of rumors based on 2 dimensions of users (age and time), susceptibility based on education classes, control strategies to effectively restrain rumor propagation, and numerical simulations to verify the main theoretical results. The study concluded that improving education levels and conducting short-term online education are essential strategies for effectively controlling rumor spread. In addition, Pulido et al [46] focused on the social impact of research to identify types of false health information shared on social media (Reddit, Facebook, and Twitter) using the application of social impact in social media (SISM) methodology. The results indicated that messages focusing on fake health information are primarily aggressive, while those based on the evidence of social impact are respectful and transformative, and deliberation contexts promoted on social media overcome false health information. The findings provide insights into how public health initiatives can support the presence and interactions of evidence as an effective strategy to combat fake news.

A study by Kim and Kim [47] investigated the misinformation belief produced in the context of COVID-19 via 2 main factors: risk perception (psychometric paradigm) and communication. It was found that perceived risk and stigma positively impact belief in fake news, while source credibility and the quantity of information reduce it. Meanwhile, among communication factors, source credibility and the quantity of information reduce belief in fake news, while the credibility of information sources increases it. In addition, Zhao et al [48] used features of online health misinformation that were classified into central level (including topic features) and peripheral level (including linguistic features, sentiment features, and user behavioral features) to propose a health misinformation detection model using the elaboration likelihood model (ELM). Based on a data set collected from a real online health community (because of the lack of a labeled data set), the model correctly detected about 85% of health misinformation. Furthermore, the findings demonstrated the efficacy of behavioral features in health misinformation detection and offered suggestions for misinformation detection by integrating the features of messages and message creators. In COVID-19–related fake news research, Wang and Huang [49] found that although an official denial can initially reduce citizens’ belief in unconfirmed information, later when the denial is revealed to be false, the citizens will have lower levels of belief, not just in the current denial, but also in the government’s future denials of similar rumors. Moreover, the negative lasting effects will carry over to satisfaction with the authorities in the related policy area.

### COVID-19 Background

This paper was initially written in 2020, and the experiment was conducted in the period from February to March 2020. Therefore, many things changed from then up to the Omicron strain of COVID-19. As such, we acknowledge that this paper has time constraints; however, the research still provides some valuable inspiration and conclusions on game studies, media development, and health care. Since the COVID-19 pandemic broke out in December 2019, the related health rumors also began to wreak havoc on the internet.

Rumor prevention is difficult in the case of rumors that rely on propaganda, and the educational means of traditional media are ineffective due to the lack of interaction and the complexity of information. On the internet, especially the midelders/elders were in a state of panic and information-blind obedience [50].

In China, an SNS group existed, in addition to many WeChat groups, similar to Discord and Facebook in the West. Therefore, we could easily find a target sample for our newly established experimental community, where any questions could be communicated at any time. Our experiment was conducted in early 2020, and some people could answer the questionnaire face to face, while others could not because of the lockdown. Therefore, some respondents were sent offline paper questionnaires, and we also requested them to fill in the online questionnaire. The Chinese midelders/elders were comfortable playing the game on their cell phones, so they easily believed the health rumor that the information communication channel is too fast. Some of them whom we could not meet face to face were contacted over a video call, and we confirmed their age and other personal information clearly to ensure accuracy in the experiment.

The original survey, questionnaire, and serious game are in Chinese, convenient for our non–English-speaking respondents, and all the concepts in this paper are the translated version. This means we just translated the statistical data and labels; during the experiment, there was no translation, and we followed the same steps for all the scales.

### Elderly WeChat Users in China: Original Survey

**Figure 1:**
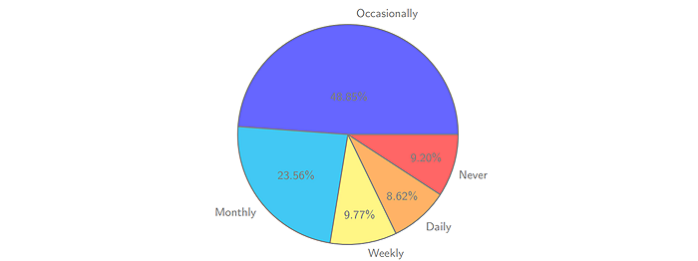
Reading frequency of SNS health information by Chinese midelders/elders. SNS: social networking service.

According to our data collected in the original survey, the contemporary middle-aged and older adults, especially those aged 40-60 years, have a high frequency of use of WeChat; the number of elderly WeChat users with frequent use accounts for 66.09% of the total. According to interviews at different levels of the questionnaire survey process, middle-aged and elderly users of WeChat are aged from 50 to 65 years. They are also familiar with using the WeChat “circle of friends” function and other social media platforms (eg, TikTok). They often record their daily lives and travel through videos and pictures. Generally, this user group is also active in online social group chats, and their frequency of using online social media is no less than that of some young user groups. For example, 48.85% of middle-aged and elderly WeChat users said they occasionally read health information on WeChat, and only 16.67% said they had never received health information forwarded by relatives and friends (Figure 1).

**Figure 2:**
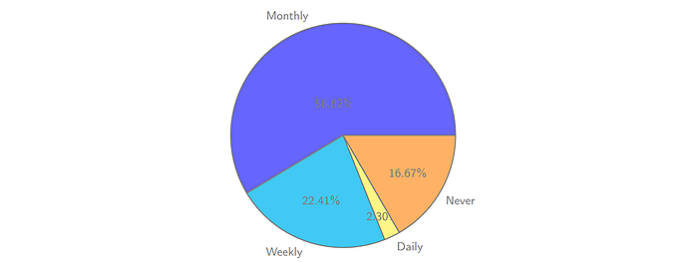
Receiving frequency of SNS health information by Chinese midelders/elders. SNS: social networking service.

The survey on the acquisition and dissemination of health information by elderly WeChat users was the focus of this study. Most people do not have the habit of reading health information regularly. It can be seen from the data that this depends to some extent on the frequency of obtaining information. People read health information only when it is forwarded to them by relatives and friends or when relevant health public accounts push this information or when it is in the form of characteristic health information news, as shown in Figure 2. Regarding access to health information, 62.07% of the respondents received health information from their WeChat friends. In addition, 83.33% of the respondents had the experience of forwarding health information to their children or parents, and 57.47% of those forwarded health information to their WeChat friends. Most respondents felt that the original intention of forwarding health information was to help others with a positive attitude.

However, many midelders/elders received health information without any judgment and recognition and then spread the information with a “good intention” motive, which is also why the health rumor issue is rampant. The data also show that the failure to recognize and identify health rumors is more likely to be the reason than the motive for spreading them. In addition, according to the questionnaire, 86.78% of elderly WeChat users trusted health information forwarded by relatives and friends and 45.4% considered it very trustworthy. The trustworthiness of health information forwarded by colleagues wa 82.18%. These data show that WeChat has become a hotbed for health rumors among the midelders/elders.

Therefore, this paper used a serious game as a tool to test the effect of game media on the prevention of health rumors. Compared with other media, the serious game had a special communication model and effect that could improve this situation (see the *Results* section for more details). Therefore, using the COVID-19 pneumonia rumor was suitable as the target and content of the serious game, involving not only the elderly closely related to COVID-19 pneumonia but also COVID-19 rumor communication relying on WeChat. Finally, the number of health rumors that emerged during the COVID-19 epidemic was enormous, and enough rumor cases could be collected for experimentation.

## Methods

### The Transmission Control Protocol Model of the Game

**Figure 3:**
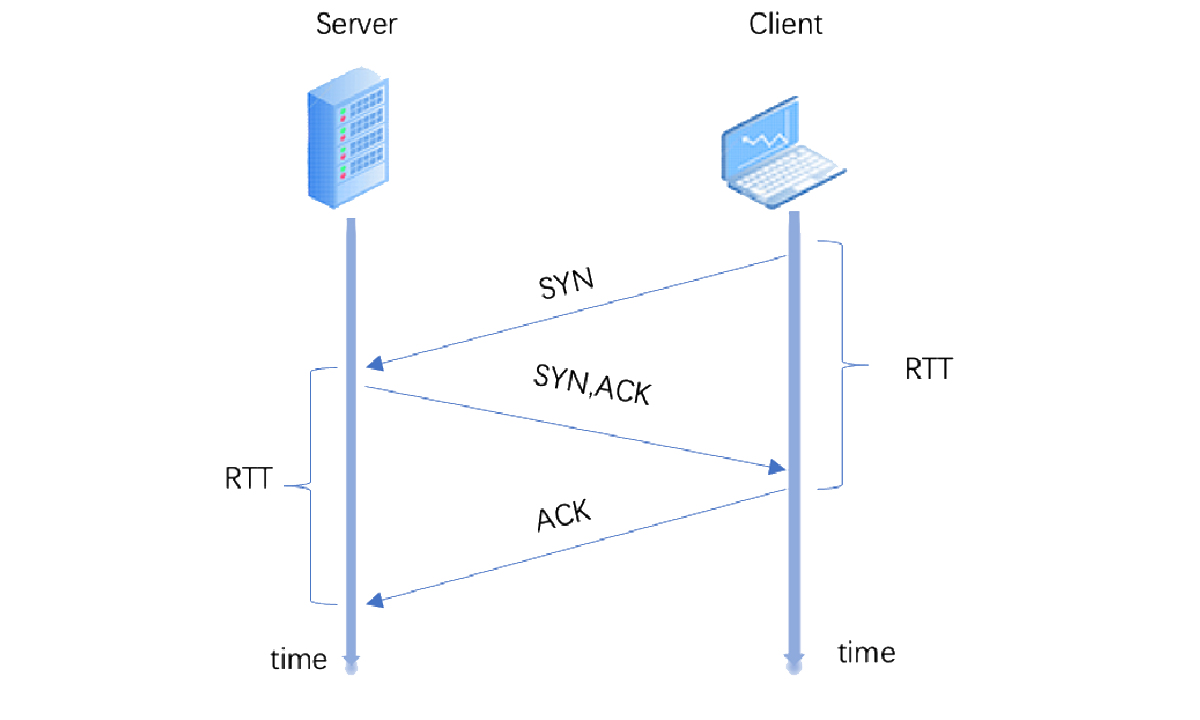
Three-way handshaking in TCP. ACK: acknowledge; RTT: round-trip time; SYN: synchronize; TCP: Transmission Control Protocol.

There are many theoretical models concerning the communication effect of games as media [51]. The computer networking concept was adopted as the inspiration for this research based on the idea of engineering. Two main protocols exist in network communication: Transmission Control Protocol (TCP) and User Datagram Protocol (UDP) [51]. TCP originated in the initial network implementation, complementing Internet Protocol (IP). TCP provides reliable, ordered, and error-checked delivery of a stream of bytes between applications running on hosts communicating via an IP network. Major internet applications, such as the World Wide Web, email, remote administration, and file transfer, rely on TCP because of the 3-way handshake mechanism (Figure 3) [52].

**Figure 4:**
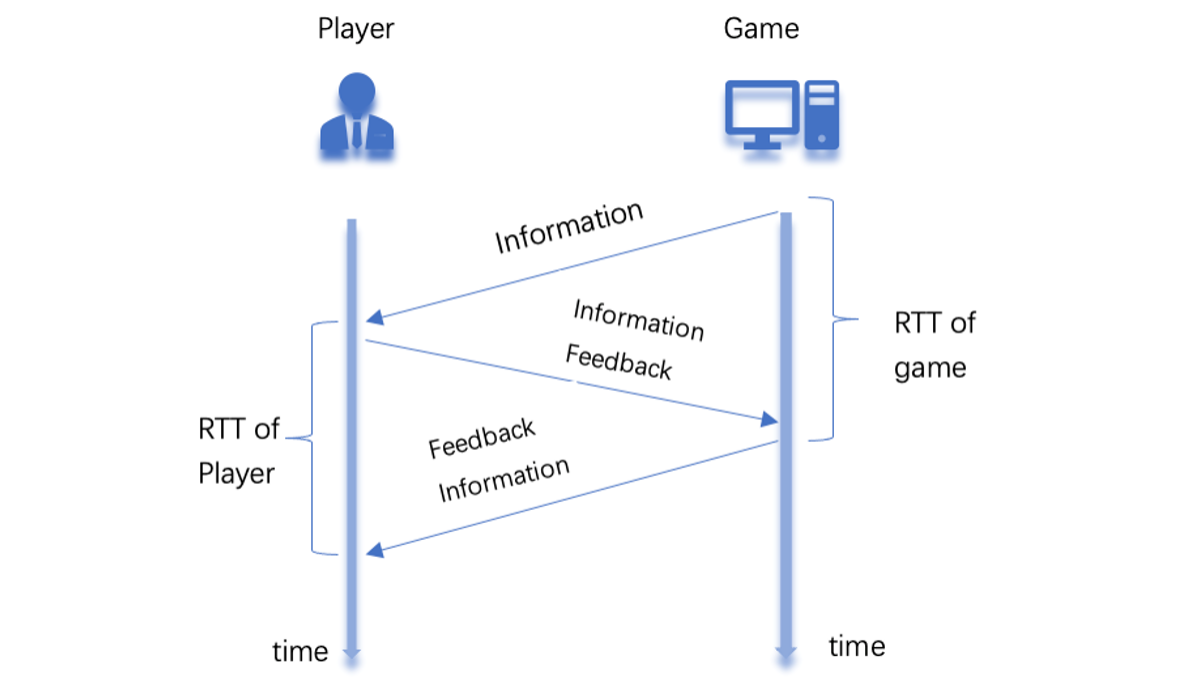
Serious game of TCP. RTT: round-trip time; TCP: Transmission Control Protocol.

Having introduced the logical mechanism of UDP and TCP from the technical perspective of communication, we can see that all media communication models are suited to TCP and UDP (2 computer network theories). UDP uses a simple connectionless communication model, just from the information source to the information sink. For example, the newspaper provides information to readers without any interaction (request and response). However, for all current media, only games match the TCP model (Figure 4). In traditional media, no matter the newspaper, broadcaster, or television program, the audience only receives information; the UDP model does not have a feedback process, the timeliness is good, but transmission is unstable. As a result, users can refuse to accept information or hardly notice useful content. Therefore, serious games can help society to address health rumor issues. In this paper, we proved the effect on the health communication area [53].

### Study Design

This study investigated the prevention and control of health rumors in WeChat, as most elderly WeChat users are concerned about health information and are negatively affected by health rumors. Here, the term “elderly” in our paper is a macroscopic definition: it is not only a physiological age classification but also a description of the psychology or state. In China, people who believe a health rumor via the SNS in the age range of 40-60 years (midelders/elders) were considered. We recruited 200 midelders/elders in Tongren City, Guizhou Province, China, which did not have a serious spread of COVID-19 in early 2020. The participants got together for dancing and training in the city plaza, and then, we requested them to attend our game experiment.

The experimental program was constructed in 2 parts. The first part was developing a serious game based on the content of health rumors and health commonsense; we named it “Fight With Virus.” The purpose was to apply this in a health rumor prevention experiment. The second part was developing a cognitive questionnaire with the theme of COVID-19 health rumor, with a 5-point Likert scale, which aimed to compare and analyze the prevention effects of traditional and serious game learning models on health rumors. Baishya and Samalia [54] extended the unified theory of acceptance and use of technology (UTAUT) into UTAUT2, incorporating 3 constructs into the original UTAUT: hedonic motivation, price value, and habit. Individual differences (ie, age, gender, and experience) were hypothesized to moderate the effects of these constructs on behavioral intention and technology use, thus affecting their learning of new technologies. Therefore, according to several past studies based on the UTAUT2 model [55,56], this study adopted the UTAUT2 model to analyze the effect of the serious game. We modified and added variables, which were analyzed using IBM SPSS Amos and IBM SPSS Statistics on factors influencing health information use and dissemination. On this basis, a suitable serious game experiment scheme was built.

The experiment was conducted in 4 steps. In steps 1 and 2, we selected the target participants (midelders/elders), while in steps 3 and 4, we designed the game for the experiment.

#### Step 1

The construction of the experimental program based on serious games and experimental research needed to be based on a full understanding of the use and dissemination of health information by the research participants. We analyzed the health information needs of the research participants, the frequency and channels of use and the dissemination of health information, and their ability to identify and judge health rumors.

#### Step 2

To investigate the phenomenon of the dissemination of health information in WeChat’s midelder/elder user groups, we used a questionnaire designed in 3 parts: The first part involved a survey to collect personal information, such as gender, age, place of residence, income level, and education. The second part was a survey on the habit of using WeChat. The third part mainly involved the frequency, channel, and motivation of users to obtain and forward health information.

At the same time, 30 health rumor judgment questions were attached to this survey questionnaire, and respondents were asked to judge whether they were correct or incorrect. Through the correct rate of health rumor judgment, we determined the trust level and ability of the respondents to identify health rumors. We also popularized the 30 relevant health rumors, with the hope to popularize the degree of health rumor knowledge and also to strengthen the respondents’ ability to recognize information. The questionnaire is shown in Multimedia Appendix 1.

Based on the cognitive ability determined through the questionnaire, 200 participants were selected and asked for their willingness to play the serious game.

#### Step 3

Based on the use and dissemination of health information by the research participants, the theme of the health rumor learning content was selected and a serious game experimental scheme suitable for this group was constructed through the design and production of serious game content. Considering the experimental length of the serious game and the understanding and acceptance level of the participants, the video game mode of a multiline plot was not applicable for our research, so a single-line plot and scenario was used in the design of the game.

**Figure 5:**
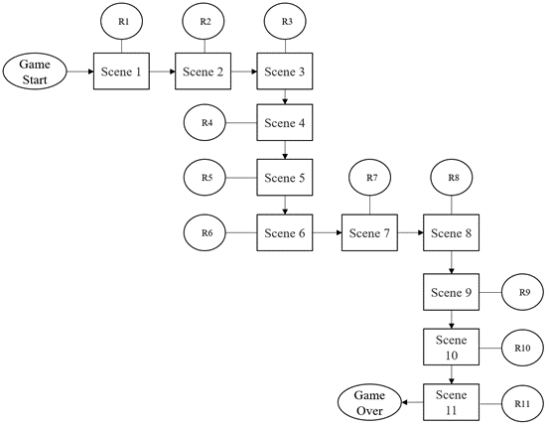
The game process. R: rumor.

**Figure 6:**
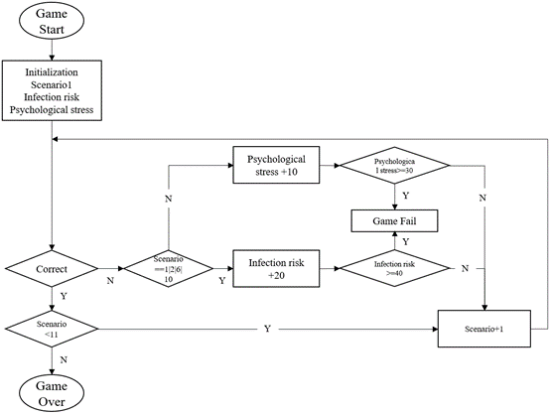
The game judgment logic. N: no; Y: yes.

The learning content of the serious game is mainly based on the theme of “a personal day,” and the content of the game plot is a person’s life from morning to afternoon, in the form of a single storyline. An explanation is provided at the beginning of the game to accurately communicate the theme, rules, and intent of the game to the players. In the learning content of the game, information such as health rumors and general knowledge about COVID-19 was selected, as shown in Table 1, and based on the selected content, failure/passing conditions were set for the game, which involved “risk of infection” and “psychological stress.” Different scenarios are set up in a day’s life, and questions are set up to interact with the game players to promote and increase the knowledge of COVID-19-related rumors in this interactive learning serious game. The main line design is shown in Figure 5, and the game logic is shown in Figure 6.

In this study, to achieve the effect of the serious game and the purpose of health rumor prevention, a feedback link of the serious game–based health rumor control prevention experiment was important. The feedback link was mainly achieved by setting up a feedback mechanism, which reflected the understanding of the research participants (players) of the game content (COVID-19 health rumor); by setting up the feedback mechanism, interactivity with the research participants could also be strengthened. At the same time, the feedback data were used to reflect the learning effect of the serious game.

The feedback mechanism of the serious game–based health rumor prevention experiment was implemented in the following 3 parts.

- The first part was to communicate the theme, rules, and intention of the game to the research participants by means of game instructions at the beginning of the game. This is an important step to quickly integrate the player into the learning process of a serious game and to let the player know what they will do next in the game.
- The second part was realized in the textual feedback of the gameplay process, where the player was provided with choices through interactive video scenarios, and instant feedback was provided. Instant feedback is an important part of the overall feedback process, which needs to be clearly communicated to the player. It is necessary to clearly communicate to the player whether their choices are correct and to strengthen the knowledge of health rumors and general health. The textual feedback content of the game process is shown inTable 2.
- The last part was to provide feedback after the player passed or failed in the game. At the end of the serious game, based on the player’s overall understanding of COVID-19 health rumors and health knowledge, the feedback can strengthen the player’s knowledge of health rumors.

**Figure 7:**
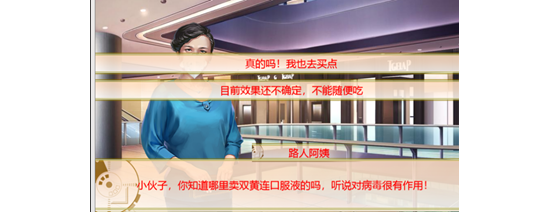
The experimental serious game’s gameplay content 1 (Chinese version). Question: “Hi boy, do you know where one can buy Shuanghuanglian Oral Liquid (a Chinese medicine)? I hear it is useful for COVID-19 treatment!” Answer options: (A) “Really? I also want to buy some.” (B) “We do not know the drug’s action, so do not drink it by yourself!”.

**Figure 8:**
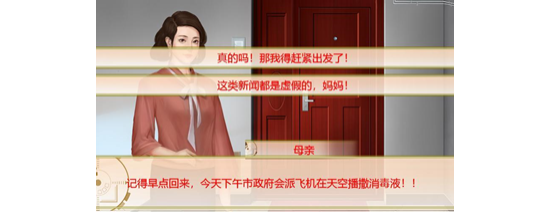
The experimental serious game’s gameplay content 2 (Chinese version). Question: “Please come back to home soon; the government will use military aircraft to spray disinfectants!” Answer options: (A) “Really? I’m leaving right now.” (B) “Fake news, Mom!”.

**Figure 9:**
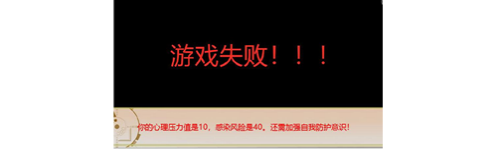
The experimental serious game: game over (Chinese version). Meaning: “The psychological pressure is 10, the risk of infection is 40, you are in a high-risk situation!”.

**Figure 10:**
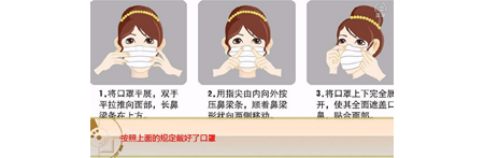
The preventive knowledge statement after the game, explaining how to wear a mask in 3 steps.

**Figure 11:**
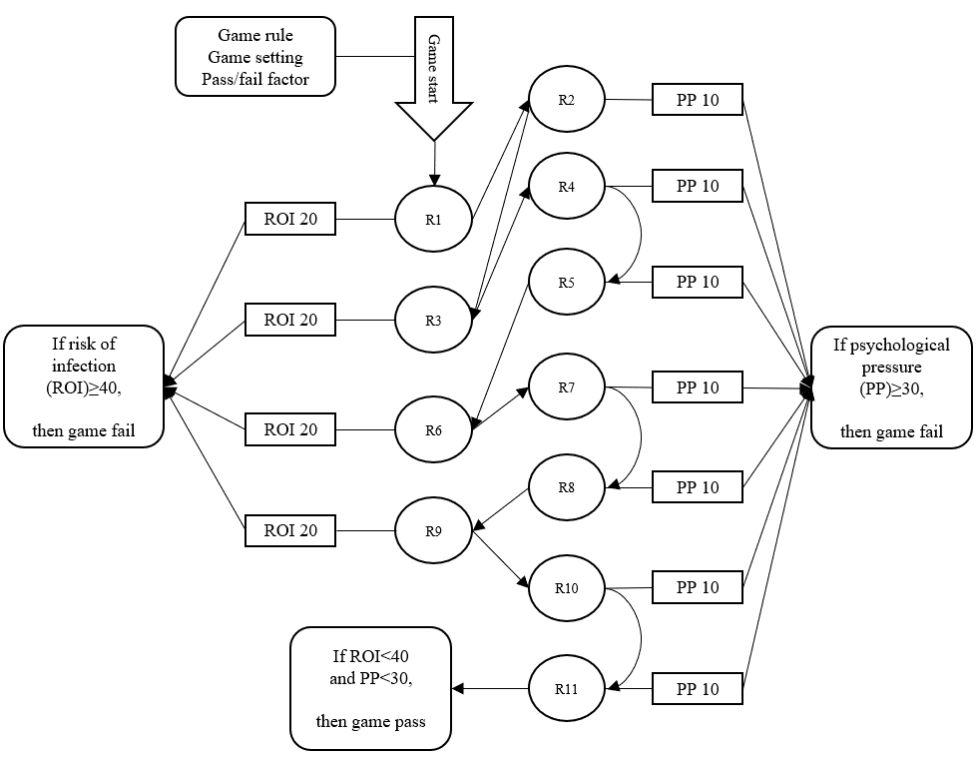
Game-related data values and failure/passing condition-setting thinking diagram.

The serious game created in this study used a COVID-19 health rumor as the learning content (see Figures 7 and 8). To achieve the purpose of preventing and controlling health rumors, a textual feedback mechanism was designed, involving 4 infection risks and 7 psychological stress settings. These were assigned to game failure or passing conditions, as shown in Figures 9 and 10. The game data reflected the performance of the research participant (player) in the game, with the settings shown in Table 3 to cater to the experiment’s needs. The specific game data value settings and game passing/failure conditions are shown in Figure 11. Specifically, the story is as follows: The protagonist, a young person, suddenly finds themselves caught up in the COVID-19 pandemic in early 2020 in their city. Various pieces of information related to COVID-19 start to emerge around the protagonist, causing a massive explosion of fear and panic, particularly among many elderly people who turn to social media for information. They begin to demand that the protagonist follow their advice on preventing the pandemic. The goal of the game is to distinguish between real health knowledge and rumors throughout the daily life story, to use accurate knowledge to save the elderly citizens who are in a state of panic, and to slow down the spread of the virus. In the end, the game outcome is judged based on the actions and choices made by the protagonist.

**Table 1:** Selection of health rumors/health facts for the serious game content.

Time of day and COVID-19 health rumor/health fact		Setting
**Morning**		
	R^a^1: Drinking plenty of boiled water at 60°C can prevent COVID-19.	Infection risk
	R2: Domesticated dogs and cats can also spread COVID-19.	Psychological stress
	R3: Putting used masks in a sterilizer can continue to provide protection and use.	Infection risk
	The correct way to wear a mask (not an option).	General health knowledge
	R4: Going out with ginger slices in the mouth can prevent COVID-19.	Psychological stress
	R5: The government will use military aircraft to spread disinfectants in the sky.	Psychological stress
**Noon**		
	R6: You should keep more than 1 m distance from strangers when you go out in times of an epidemic.	Infection risk
	R7: Eye-to-eye contact may transmit COVID-19.	Psychological stress
	R8: Shuanghuanglian Oral Liquid can effectively inhibit the COVID-19 virus.	Psychological stress
**Afternoon**		
	R9: Disinfection is required for items after returning home from outside.	Infection risk
	R10: High temperature can kill the virus, so hot blow-drying and hot water bathing can inhibit it.	Psychological stress
	R11: Do not eat fish; pickled fish made from grass carp can transmit COVID-19.	Psychological stress

^a^R: rumor.

**Table 2:** Text feedback during gameplay.

Rumor number	Textual feedback (explanation and education) of scenario options during gameplay
R^a^1	Drinking water does not help, and scalding the mucous membrane of the mouth with hot water can increase the risk of infection.
R2	There is no evidence that COVID-19 can be transmitted to domesticated dogs and cats.
R3	Masks that have been used many times do not work to isolate droplets.
—^b^	Graphic feedback: follow the 3 steps (regulations) to wear the mask correctly.
R4	Ginger does not work to prevent the COVID-19.
R5	This is a rumor. There are no military aircraft to spread disinfectants in the sky; in addition, the local government has no right to do that. (This raises the players’ sense of alertness and achieves the purpose of public education.)
R6	COVID-19 is spread via droplets and contact, and close contact increases the risk of infection.
R7	This is a rumor. The virus is transmitted through bodily fluids, droplets, and aerosols, not through the eyes. (This re-explains the mode of transmission of the COVID-19 virus.)
R8	Clearly inform that this is not yet clear information and should not be followed blindly.
R9	This is true. You should do that. (This provides possible contact transmission and health information on sterilization.)
R10	Dizziness and other symptoms can occur if you are exposed to bath bombs for too long or take a hot bath for too long.
R11	COVID-19 only infects mammals. Fish do not transmit COVID-19.

^a^R: rumor.

^b^Not applicable.

**Table 3:** Game data and game rating settings.

Game score	Error choice	Game failure	Game round
A	≤3	0	1
B	4-6	1	2
C	7-9	2	3
D	≥10	≥3	≥4

#### Step 4

Finally, the data and feedback of the participants were obtained, and the effect of the serious game on health rumor prevention was analyzed through the data and feedback to determine whether serious games are useful to prevent health rumors.

### Data Collected

This study compared and analyzed the differences between acquiring and understanding health rumor information through the learning modes of serious games and traditional media. A total of 100 people were selected to participate in the serious game experiment (G1 group), while 100 people who did not participate in the serious game experiment only studied by traditional media (G2 group). To ensure the objectivity of the controlled experiment, the educational background of the 200 participants was investigated before the formal study while keeping the 2 groups as similar as possible in terms of gender and age, as Table 4 shows. Next, we sent the testing questionnaire related to health commonsense and health rumors to G1 and G2.

**Table 4:** Demographic information of groups G1 and G2.

Characteristics		G1 (n=100), n (%)	G2 (n=100), n (%)
**Gender**			
	Male	42 (42)	40 (40)
	Female	58 (58)	60 (60)
**Age (years)**			
	40-45	24 (24)	27 (27)
	46-50	30 (30)	25 (25)
	51-55	19 (19)	17 (17)
	56-60	18 (18)	21 (21)
	≥61	9 (9)	10 (10)
**Educational background**			
	Less than high school	15 (15)	15 (15)
	High school/technical secondary school	50 (50)	50 (50)
	Junior college	20 (20)	20 (20)
	Bachelor’s degree and higher	15 (15)	15 (15)
**Income**			
	Low	29 (29)	24 (24)
	Average	48 (48)	55 (55)
	High	23 (23)	21 (21)

### Variables and Hypotheses

**Figure 12:**
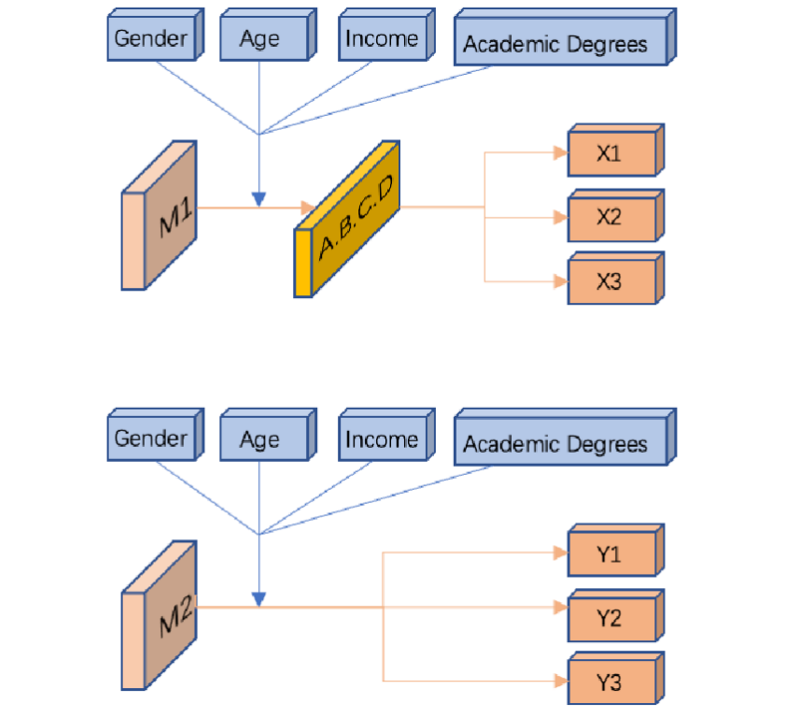
Relationship between experimental study variables.

Based on the framework of serious games, a questionnaire was designed that included the following variable definitions:

- Independent variables: The learning mode refers to the way that information is obtained and knowledge learned; the variables were M1 (learning through serious games) and M2 (learning through traditional and new media). For gender, age, income, and education, in the preparation stage of the research, education was used as the main grouping basis, and the age distribution and gender ratio of the 2 groups of experimental objects were kept consistent.
- Intermediary variables: Game data for serious game experiments (A/B/C/D) refer to the player’s performance data during the game, including the number of selection errors, the number of game failures, and the number of game passings. Personal performance was divided into 4 mediating variables: excellent (A), good (B), medium (C), and poor (D).
- Dependent variables: The variables were cognitive questionnaire (1) overall correct response rate of judgment and recognition of the COVID-19 health rumor (X1 for G1, Y1 for G2 [G2 did not participate in the serious game experiment]), (2) correct rate of judgment and recognition the COVID-19 health rumor part 1 (X2 for G1 [COVID-19 health rumor not included in the serious game experiment], Y2 for G2), and (3) correct rate of judgment and identification of the COVID-19 health rumor part 2 (X3 for G1, Y3 for G2 [COVID-19 pneumonia rumor included in the traditional media experiment for G2]).
- Intervening variables: The comprehension, cognitive level, learning ability, learning interest, and information attention of the G1 group affected the outcome of the dependent variables to a certain extent. The specific influencing relationship between various variables is shown in Figure 12.

The following 10 hypotheses were proposed in this experimental study:

- Hypothesis 1 (H1): Serious game experiments can help research participants acquire and understand health rumor knowledge and health commonsense.
- H2: The serious game learning mode is more capable of inspiring the interest of research participants in their understanding of the knowledge acquired and their learning of health commonsense compared to the traditional learning mode.
- H3: The serious game learning mode is more impactful than the traditional learning mode.
- H4: In judging and recognizing the COVID-19 health rumor, G1 has a stronger judgment ability than G2 and a higher accuracy in identifying the rumor in the serious game experiment.
- H5: In judging the COVID-19 health rumor, for the rumor not included in the serious game experiment, without the influence of M1, the ability of G1 and G2 is not much different, and the accuracy rate of identifying the COVID-19 health rumor is roughly the same for both groups. The manifestation in the variable is X2≈Y2.
- H6: In judging and recognizing the COVID-19 health rumor, G1 has an overall stronger judgment ability than G2 and a higher accuracy rate of identifying the COVID-19 health rumor. The specific manifestation in the variable is X3>Y3.
- H7: Gender affects G1’s and G2’s judgment and recognition of the COVID-19 health rumor.
- H8: Age affects G1’s and G2’s judgment and recognition of the COVID-19 health rumor.
- H9: Income affects G1’s and G2’s judgment and recognition of the COVID-19 health rumor.
- H10: Academic qualifications affect G1’s and G2’s judgment and recognition of the COVID-19 health rumor.

### Ethical Considerations

According to the guidelines of the People’s Republic of China [57], this study met the conditions for exemption from ethical review.

## Results

### Analysis of Data Collected

**Figure 13:**
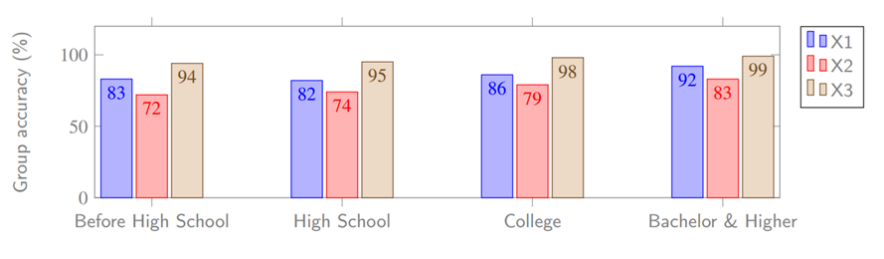
Comparison of corresponding dependent variables (G1).

**Figure 14:**
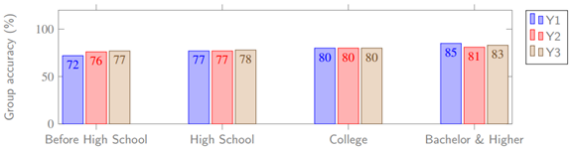
Comparison of two groups and corresponding dependent variables (G2).

According to the collected game data, 36% (72/200) of players received A, 41% (82/200) received B, 18% (36/200) received C, and the remaining 5% (10/200) received D. The accuracy rate of the judgment and recognition of the COVID-19 health rumor and health commonsense in the cognitive questionnaire of G1 and G2 groups are tabulated in Table 5. The overall accuracy was 84% for G1 and 78% for G2, with an average of 81%. The relationship of the parameters X1-X3 (G1) and Y1-Y3 (G2) are shown in Figures 13 and 14, respectively.

**Table 5:** Cognitive questionnaire data (judgment and recognition of rumor knowledge and health commonsense).^a^

Question number	Accuracy G1 (%)	Accuracy G2 (%)	Average accuracy (%), (G1+G2)/2
1	72	76	74
2	93	98	96
3	75	79	77
4	77	76	77
5	64	63	64
6	87	91	89
7	77	85	81
8	72	70	71
9	67	63	65
10	80	74	77
11	67	64	66
12	80	88	84
13	88	96	92
14	75	71	73
15	81	83	82
16	91	95	93
17	68	63	66
18	80	83	82
19	58	56	57
20	97	71	84
21	99	61	80
22	98	65	82
23	99	92	96
24	91	81	86
25	100	92	96
26	100	89	95
27	90	76	83
28	92	85	89
29	92	67	80
30	99	95	97

^a^All the correct rate values are kept to integer bits.

According to the data, the overall average correct rate of judgment and recognition of G1 and G2 was 84% and 78%, respectively. In addition, the related data collected showed that 76% of the participants believed that the serious game learning mode is more interesting than the traditional learning mode. This finding shows that G1 has good interest in games. Furthermore, 60% of the participants thought that the game learning mode is more helpful than the traditional learning mode, and 65% thought that the serious game learning mode makes a more profound impression. Therefore, from the perspective of the selection of participants, the serious game learning mode is more interesting, helpful, and impactful than the traditional learning mode. Therefore, H2 and H3 hold.

### Analysis of Dependent Variables (X, Y) and Intermediary Variables (A, B, C, D)

According to the data of the dependent variables in Table 6, we could not directly prove that the impact of M1 on X1 was greater than that of M2 on Y1. At the same time, when the knowledge of health rumors and health commonsense was not included in the game content, the correct rate of judgment and recognition of G1 and G2 was almost the same, and even G1 had a relatively lower rate. However, X3 was 96%, which is much higher than Y3 (79%), and X3 exceeded X2 by up to 23 percentage points. This condition implies that the impact of M1 on X1 is greater than the impact of M2 on Y1. It not only shows that G1 had relatively strong learning ability but also that after the serious game learning model experiment, there was a significant positive effect on the accuracy rate of the judgment and recognition accuracy of the COVID-19 health rumor and health commonsense. At the same time, X1>Y1, X2≈Y2, and X3 >Y3. Therefore, H4, H5, and H6 are established.

The intermediary variables A, B, C, and D were sequentially observed, corresponding to the dependent variables X1, X2, and X3. It can be clearly seen in Table 7 that A-X1>B-X1>C- X1>D-X1, A-X2>B-X2>C-X2>D-X2, and A-X3>B-X3>C-X3>D-X3. The dependent variables corresponding to the intermediary variables showed a decreasing trend from A to D, indicating that M1 affected X1 and X3 through A, B, C, and D and the degree of influence was in the order of A>B>C>D. These data showed that the higher the average game score, the higher the correct rate of recognition and judgment. Therefore, combined with the previous analysis, H1 holds.

**Table 6:** Dependent variables X and Y.

Variable	Value (%)
X1^a^	84
X2^b^	76
X3^c^	96
Y1^d^	78
Y2^e^	78
Y3^f^	79

^a^X1: cognitive questionnaire overall correct response rate of judgment and recognition of the COVID-19 health rumor for G1.

^b^X2: cognitive questionnaire correct rate of judgment and recognition part 1 for G1.

^c^X3: cognitive questionnaire correct rate of judgment and identification of the COVID-19 health rumor part 2 for G1.

^d^Y1: cognitive questionnaire overall correct response rate of judgment and recognition of the COVID-19 health rumor for G2.

^e^Y2: cognitive questionnaire correct rate of judgment and recognition part 1 for G2.

^f^Y3: cognitive questionnaire correct rate of judgment and identification of the COVID-19 health rumor part 2 for G2.

**Table 7:** Intermediary variables (A, B, C, D) and dependent variables (X1-X3).

Intermediary variable	Dependent variables		
	X1^a^ (%)	X2^b^ (%)	X3^c^ (%)
A	87	80	98
B	83	76	96
C	80	72	94
D	72	62	89

^a^X1: cognitive questionnaire overall correct response rate of judgment and recognition of the COVID-19 health rumor for G1.

^b^X2: cognitive questionnaire correct rate of judgment and recognition part 1 for G1.

^c^X3: cognitive questionnaire correct rate of judgment and identification of the COVID-19 health rumor part 2 for G1.

### Analysis of Independent Variables

#### Gender

We grouped participants, ensuring the educational composition of the 2 groups was as consistent as possible. By observing and comparing the G1 independent variable (gender) and its corresponding intermediary variables (Table 8), we found that the game score reached A, where the male participants were better than the female participants but changed from B to D when the female participants were better than the male participants. This condition was especially true when the game score reached B, where the female participants were much better than the male participants. This situation may also be influenced by the unequal relationship of the overall gender. There was no gender difference in the numbers from game rating A to D.

Subsequently, by observing and comparing the G1 and G2 independent variable gender and the corresponding dependent variables (Table 9), we observed that regarding the dependent variables X1, X2, and X3, corresponding to the independent variable gender (female, male), the comparisons were female<male, female<male, and female>male, respectively. Regarding Y1, Y2, and Y3, corresponding to gender, the comparisons were female<male, female>male, and female<male, respectively. As such, there was no gender difference. Therefore, H7 does not hold.

**Table 8:** G1 gender and corresponding intermediary variables.

Intermediary variable	Gender		Comparison
	Female	Male	
A	16	20	Female<male
B	27	14	Female>male
C	12	6	Female>male
D	4	1	Female>male

**Table 9:** G1 and G2 gender and corresponding dependent variables.

Dependent variable	Gender		Comparison
	Female (%)	Male (%)	
X1^a^	83	84	Female<male
X2^b^	75	77	Female<male
X3^c^	97	95	Female>male
Y1^d^	76	81	Female<male
Y2^e^	79	77	Female>male
Y3^f^	71	88	Female<male

^a^X1: cognitive questionnaire overall correct response rate of judgment and recognition of the COVID-19 health rumor for G1.

^b^X2: cognitive questionnaire correct rate of judgment and recognition part 1 for G1.

^c^X3: cognitive questionnaire correct rate of judgment and identification of the COVID-19 health rumor part 2 for G1.

^d^Y1: cognitive questionnaire overall correct response rate of judgment and recognition of the COVID-19 health rumor for G2.

^e^Y2: cognitive questionnaire correct rate of judgment and recognition part 1 for G2.

^f^Y3: cognitive questionnaire correct rate of judgment and identification of the COVID-19 health rumor part 2 for G2.

#### Age

There were apparent differences in the age ranges between the 2 groups, as shown in Table 4. Therefore, random sampling in G1 and G2 was conducted, and 25 participants under 51 and 56 years old each were selected, with 50 participants in each group for comparative observation and analysis of the corresponding variable data.

First, by observing and comparing the high-age and low-age groups’ independent and variable age groups and their corresponding intermediary variables (Table 10), we found that the number of people who achieved A and B game scores were all of low age. As a result, the number of people in the low-age group was greater than the number of people in the high-age group; among those with game scores C and D, the number of people in the high-age group was greater than the number of people in the low-age groups, indicating that to a certain extent, the independent variable age positively affects the intermediary variables A, B, C, and D. Second, by observing and comparing the high- and low-age groups of the G1 and G2 independent variable age with corresponding dependent variables (Table 11), the values of independent variables X1, X2, and X3 could be determined. The values of the low-age group were greater than those of the high-age group; the independent variables Y1, Y2, and Y3 also exhibited this behavior. Therefore, H8 holds.

**Table 10:** G1 and G2 targets of different ages.

Intermediary variable	Age	
	Low (n=25), n (%)	High (n=25), n (%)
A	8 (32)	13 (52)
B	6 (24)	8 (32)
C	8 (32)	3 (12)
D	3 (12)	1 (4)

**Table 11:** G1 and G2 age groups and corresponding dependent variables.

Dependent variable	Age	
	Low (%)	High (%)
X1^a^	76	87
X2^b^	69	80
X3^c^	88	98
Y1^d^	68	82
Y2^e^	66	80
Y3^f^	72	86

^a^X1: cognitive questionnaire overall correct response rate of judgment and recognition of the COVID-19 health rumor for G1.

^b^X2: cognitive questionnaire correct rate of judgment and recognition part 1 for G1.

^c^X3: cognitive questionnaire correct rate of judgment and identification of the COVID-19 health rumor part 2 for G1.

^d^Y1: cognitive questionnaire overall correct response rate of judgment and recognition of the COVID-19 health rumor for G2.

^e^Y2: cognitive questionnaire correct rate of judgment and recognition part 1 for G2.

^f^Y3: cognitive questionnaire correct rate of judgment and identification of the COVID-19 health rumor part 2 for G2.

#### Income

The relevant data collected are shown in Table 4. Nearly half of the participants in G1 and G2 believed that their income level was average, and the number of people who believed that their income was high or low was relatively small. By observing and comparing the high- and low-income subgroups in G1 and G2 with corresponding dependent variables, we found that the dependent variables corresponding to the 2 independent variable subgroups were not identical, as shown in Table 12. Therefore, H9 does not hold.

**Table 12:** High- and low-income groups and corresponding dependent variables of G1 and G2.

Dependent variable	Income	
	High (%)	Low (%)
X1^a^	84	84
X2^b^	82	78
X3^c^	88	94
Y1^d^	73	74
Y2^e^	69	72
Y3^f^	80	78

^a^X1: cognitive questionnaire overall correct response rate of judgment and recognition of the COVID-19 health rumor for G1.

^b^X2: cognitive questionnaire correct rate of judgment and recognition part 1 for G1.

^c^X3: cognitive questionnaire correct rate of judgment and identification of the COVID-19 health rumor part 2 for G1.

^d^Y1: cognitive questionnaire overall correct response rate of judgment and recognition of the COVID-19 health rumor for G2.

^e^Y2: cognitive questionnaire correct rate of judgment and recognition part 1 for G2.

^f^Y3: cognitive questionnaire correct rate of judgment and identification of the COVID-19 health rumor part 2 for G2.

#### Education

Based on the cognitive questionnaire, the independent variable education was divided into 4 segments, as shown in Table 4. First, 4 groups of the G1 independent variable education and corresponding intermediary variables were compared (Table 13). Through comparison and observation, we found that the higher the education level, the better the performance in the game, which demonstrates that the dependent variable education positively affects the intermediary variables. Second, the grouping and corresponding dependent variables of G1 and G2 based on academic qualifications are shown in Table 14. We found that education has a positive effect on the corresponding dependent variables. Therefore, H10 holds.

**Figure 15:**
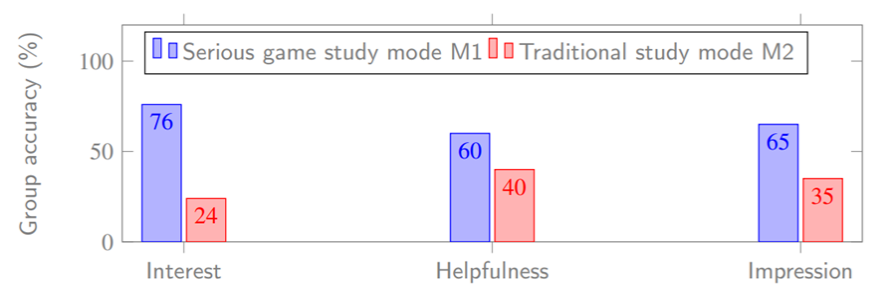
Comparison between serious game learning mode and traditional learning mode.

Finally, a thorough investigation of the selection tendency between the learning modes of serious games (M1) and traditional learning (M2) was conducted on G1 involving a comparison experiment of interest, help, and impression (Figure 15). The data showed that 76% (152/200) of the participants thought the serious game learning mode was more interesting than the traditional learning mode, indicating that G1 had a reasonable learning interest in games. Furthermore, 60% (120/200) of the participants thought that the serious game learning mode was more helpful than the traditional learning mode, and 65% (130/200) thought that the serious game learning mode was more impressive than the traditional learning mode. Therefore, from the perspective of the selection tendency of the participants, the serious game learning mode is more interesting, helpful, and impressive than the traditional learning mode, which strengthens the hypotheses.

**Table 13:** Proportion of game scores in different education segments.

Segment	Intermediary variables			
	A (%)	B (%)	C (%)	D (%)
Less than high school	20	47	20	13
High school	28	44	20	8
College degree	45	40	15	0
Bachelor’s degree and higher	59	23	13	0

**Table 14:** Education segments and corresponding dependent variables of G1 and G2.

Segment	Dependent variables					
	X1^a^ (%)	X2^b^ (%)	X3^c^ (%)	Y1^d^ (%)	Y2^e^ (%)	Y3^f^ (%)
Less than high school	83	72	94	72	76	77
High school	82	74	95	77	77	78
College degree	86	79	98	80	80	80
Bachelor’s degree and higher	92	83	99	85	81	83

^a^X1: cognitive questionnaire overall correct response rate of judgment and recognition of the COVID-19 health rumor for G1.

^b^X2: cognitive questionnaire correct rate of judgment and recognition part 1 for G1.

^c^X3: cognitive questionnaire correct rate of judgment and identification of the COVID-19 health rumor part 2 for G1.

^d^Y1: cognitive questionnaire overall correct response rate of judgment and recognition of the COVID-19 health rumor for G2.

^e^Y2: cognitive questionnaire correct rate of judgment and recognition part 1 for G2.

^f^Y3: cognitive questionnaire correct rate of judgment and identification of the COVID-19 health rumor part 2 for G2.

## Discussion

### Principal Findings

Based on a self-made serious game, this paper investigated the health rumor phenomenon, and a study on the user behavior and willingness to disseminate health information among Chinese elderly WeChat users (SNS) was conducted during the early COVID-19 pandemic. After a survey, participants were chosen, and a COVID-19 health rumor was selected as the study content and the experimental platform with the self-made game was established. The UTAUT2 model was upgraded by adding parameters, several hypotheses were proposed, and a control experiment was designed. The experiment results show that the serious game is useful for health rumor prevention.

After collecting game data and the correct response rates of G1 and G2 in the cognitive questionnaire for the judgment and recognition of the COVID-19 health rumor, the game data and the cognitive questionnaire data were combined to determine the relationship between specific variables. Finally, the experimental hypotheses were tested and evaluated, proving that H1-H6, H8, and H10 hold, while H7 and H9 do not hold.

The findings affirm that serious games are a powerful tool to enhance learning and commonsense against health rumors in the context of elderly users of SNS. As Wu [43] argued, perceptions of rumor credibility affect the users’ desire to find accurate information (*cognitive gratification*) because they use SNS for verifying the contents of rumors and for acquiring more knowledge and information. Equipping oneself with better knowledge and commonsense against health rumors could have a profound effect on the stability and harmony of society [58], minimize the chance of being misinformed [35], and help create effective control strategies against rumor spreading [45]. Furthermore, as serious games provide the means for people to receive direct feedback relative to their judgment of health rumors, using these games is considered a more humane and emotional approach [47]. In addition, it also provides a suitable channel for health care providers to increase awareness [49] since tackling COVID-19 requires everyone to follow medical advice. Based on the verification of our hypotheses, we found that the effect of serious games correlates with parameters such as education, which suggests that the future rumor management for the youth is perfectly suited to the use of serious games, especially in China, where the education level of the youth is much higher than that of the middle-aged and older populations.

### Limitations

Given the seminal findings of this study, it has some limitations. First, the cognitive questionnaire was administered offline, and the midelder/elder participants were reluctant in terms of their willingness to cooperate with the research. As such, there was a risk of the sample distribution being uneven or biased. Second, strict epidemic prevention and control have geographically limited experimental samples. Third, the serious game design was restricted to the COVID-19 health information and had limited interactivity.

### Conclusion

This experimental study on preventing new health rumors via serious games proves that the serious game learning mode can help research participants understand and learn about health and rumors. Furthermore, serious games make a more profound impression on people than traditional learning modes, while providing fertile ground for more comprehensive research in the future. In addition, serious games could provide suggestions and support in future research on rumor prevention and detection. In particular, the Chinese government ended the zero-COVID policy in December 2022, and many new health rumors related to the Omicron variant were found on the internet in China. This study could provide a method of challenging the new issue and the game could be updated for the current situation. More importantly, we discovered that serious games can act as an “informational vaccine” against rumors (if rumors are considered a kind of “informational virus or bacterium”), and in the future, we can conduct further research in this direction.

## Appendix

Multimedia Appendix 1 A priori questionnaire for health rumor recognition.

## Abbreviations

GM: genetically modified
HCW: health care worker
IP: Internet Protocol
SNS: social networking service
TCP: Transmission Control Protocol
UDP: User Datagram Protocol
UTAUT: unified theory of acceptance and use of technology
