# Supplementary material for: A Serious Game (“Fight With Virus”) for Preventing COVID-19 Health Rumors: Development and Experimental Study
Source: JMIR Serious Games. 2024 Feb 26;12:e45546. doi: 10.2196/45546 (PMC10936928; doi:10.2196/45546)
Supplement: Multimedia Appendix 1 [file games_v12i1e45546_app1.docx]

**Multimedia Appendix 1.** The a priori questionnaire for health rumor recognition

| No | Questions |
| --- | --- |
| 1 | Your gender |
| 2 | Your age |
| 3 | Your place of residence |
| 4 | Your income level within your place of residence |
| 5 | Your education level |
| 6 | How often do you use WeChat in your daily life? |
| 7 | In your daily life, how often do you perform the following behaviors on WeChat? (Matrix) |
| 8 | In your daily life, do you read relevant health and wellness articles on WeChat? (Multiple choice) |
| 9 | Do you think the relevant health and wellness information you receive on WeChat is helpful to your daily life? (Multiple choice) |
| 10 | In your daily life, do you receive information about health and wellness forwarded by your friends and relatives? |
| 11 | What do you think is the reason for you or others to forward the health and wellness information? (Multiple choice) |
| 12 | In your daily life, from which channels do you usually receive information about health and wellness? (Multiple choice) |
| 13 | Do you forward information about health and wellness in your daily life? (Matrix) |
| 14 | To whom do you forward information about health and wellness that you find credible in your daily life? |
| 15 | What are the microblogs about health and wellness that you follow in your daily life? |
| 16 | How much do you trust the health information you receive from the following channels? |
| 17 | Do you guide your behavior in life based on the health and wellness information on WeChat? |
| Survey on Recognition of Health Rumors (Judgement Questions) | |
| No | Questions |
| 1 | Bananas, persimmons, oranges, tomatoes, milk, soy milk can not be eaten on an empty stomach |
| 2 | Soak your feet in vinegar to cure foot odor |
| 3 | Repeatedly boiled water can not drink |
| 4 | Drinking coffee often can cause cancer |
| 5 | Radiation from cell phones, computers, wireless routers, TVs, microwave ovens and electromagnetic furnaces can seriously harm the human body. |
| 6 | Iodized salt can prevent radiation |
| 7 | Eating garlic every day can prevent cancer |
| 8 | Eggs are more nutritious than domesticated eggs. |
| 9 | Different colors of eggshells affect the quality of eggs |
| 10 | Chickens raised for 40 days out of the cage are harmful to human body if eaten |
| 11 | Stir-frying vegetables in an iron skillet can replenish iron |
| 12 | Bone broth and chicken broth can replenish calcium |
| 13 | Eating bird's nest can nourish the face |
| 14 | Eating walnuts can replenish the brain |
| 15 | Corn boiled in water can lower blood pressure, lower blood fat |
| 16 | Eating apples in the morning has higher nutritional value |
| 17 | Covering sweat can speed up the healing process when you have a fever |
| 18 | Spending five to six minutes a day to rotate the eyeballs can cure high myopia. |
| 19 | Ginger rubbed on hair can regrow hair |
| 20 | Eating more Colla Corii Asini, jujube, brown sugar and peanuts with red skin can replenish blood. |
| 21 | Sudden heart attack, can be used to prick the earlobe, stub toes, pinch the method to revive patients |
| 22 | Smoke vinegar in the room to prevent colds. |
| 23 | Soaking ingredients in vinegar before eating can soften blood vessels |
| 24 | Drinking salt water in the morning is good for health |
| 25 | MSG can cause cancer when heated |
| 26 | Infusion can prevent cardiovascular disease |
| 27 | Artificial eggs exist in the market |
| 28 | Some watermelons are colored red |
| 29 | Small tomatoes are genetically modified food, harmful to human body if eaten |
| 30 | Wrap the plastic wrap when exercising will lose weight faster |
